# The effects of leadership on patient safety culture in health care: a systematic review

**DOI:** 10.1186/s12912-025-04263-7

**Published:** 2026-01-08

**Authors:** Fahad M. Althobaiti

**Affiliations:** Nursing Leadership and Education Department, Nursing College, Taif University, Taif, 21974 Saudi Arabia

**Keywords:** Leadership, Patient safety culture, Health care, Systematic review, Nursing

## Abstract

**Background:**

Nurse managers or leaders adopt transformational, transactional, servant, and ethical leadership styles. Nursing leadership styles shapes patient safety in healthcare organizations. Current literature is unclear on the link between leadership styles and patient safety in hospitals. The systematic review aimed to examine the effects of leadership on patient safety culture in health care.

**Methods:**

Relevant literature was searched on PubMed, CINAHL, Cochrane Database of Systematic Reviews, ProQuest-Nursing & Allied Health, PsycINFO, and Scopus with keywords and search terms. Eligibility criteria covered studies addressing the research question, containing primary studies, written in English, and published between 2008 and 2024. PRISMA 2020 Framework was used in the screening process and data extraction conducted.

**Findings:**

Out of the 507 studies found in the databases, studies (*n* = 12) that met the eligibility criteria were included. Quality appraisal of the studies showed low risk of bias and hence their inclusion for narrative synthesis. Data extracted and subsequent narrative synthesis found that transformational leadership, ethical leadership, and transactional leadership styles, which impacted patient safety culture positively improved teamwork, positive manager expectations, organizational learning, feedback, communication, and positive perceptions on safety. Leadership factors affecting patient safety culture were nurses’ organizational commitment, education or training, job recourses, work engagement, and mentorship for enhancing nurses’ competence.

**Conclusion:**

The systematic review confirmed that leadership style creates patient safety culture. Future studies should examine the long-term impacts of varied leadership styles particularly transformational and ethical leadership style on patient safety in different healthcare settings.

## Introduction

Patient safety culture is widely recognized as a core component of healthcare quality because it reflects shared values, norms, and behaviors that prioritize the prevention of harm and continuous learning in clinical settings [1, 2]. Key dimensions such as communication openness, error reporting, teamwork, and organizational learning are critical for reducing adverse events including medication errors, healthcare‑associated infections, diagnostic delays, pressure injuries, and patient falls [3–5]. Despite international initiatives to strengthen safety, gaps in patient safety culture remain evident across hospitals and other healthcare organizations, indicating the need to understand modifiable organizational factors that can improve safety [6, 7].

Leadership is a central factor shaping patient safety culture because leaders influence priorities, resources, and expectations that guide how staff perceive and act on safety issues [8, 9]. In nursing and interprofessional teams, leadership behaviors such as articulating a clear safety vision, role‑modelling desired practices, supporting incident reporting, and providing feedback are closely linked to staff engagement and perceptions of safety [10, 11]. Empirical studies suggest that leadership styles including transformational, transactional, servant, and ethical leadership are associated with outcomes such as organizational commitment, teamwork, job satisfaction, and safety climate; however, findings are dispersed across different settings and measurement approaches [12–14, 15].

The existing evidence base on leadership and patient safety culture is fragmented by variation in conceptual frameworks, leadership and safety instruments, and healthcare contexts [12, 16, 17]. Many studies focus on single settings or professions, use different leadership models, or lack examination of mechanisms such as organizational commitment, job resources, work engagement, and mentorship that may mediate the relationship between leadership and safety culture [18, 19, 20]. These differences limit the ability of healthcare and nursing leaders to draw clear conclusions about which leadership approaches are most effective in strengthening patient safety culture and under what conditions [16, 21].

This systematic review aims to synthesize quantitative evidence on the effects of leadership on patient safety culture in healthcare, with particular attention to nursing and interprofessional contexts [22]. Specifically, the review examines how different leadership styles and behaviors relate to safety culture outcomes and explores mechanisms and contextual factors that may shape these relationships [12, 18, 19]. By integrating findings across diverse healthcare settings, this review seeks to generate evidence‑informed guidance for leadership development and organizational strategies that can enhance patient safety culture and, ultimately, contribute to improved health outcomes within the broader field of health sciences and nursing practice.

## Materials and methods

This systematic review was guided by PRISMA [23] to organize the methods and results. This systematic review was guided by the Preferred Reporting Items for Systematic Reviews and Meta-Analyses (PRISMA) 2020 framework 15. PRISMA is an internationally accepted set of evidence-based guidelines designed to enhance the transparency, clarity, and completeness of reporting systematic reviews and meta-analyses. The framework includes a comprehensive 27-item checklist and flow diagram that systematically direct the process of literature identification, screening, eligibility assessment, and inclusion of studies. Use of PRISMA ensures methodological rigor, reproducibility, and facilitates critical appraisal by readers and stakeholders.

The researcher selected PRISMA because it assists in systematically organizing and documenting key steps such as search strategy development, study selection, data extraction, and quality appraisal. This minimizes the risk of bias and selective reporting. The clarity brought by PRISMA improves the reliability and validity of the synthesis, thus strengthening the evidence base regarding the effects of leadership styles on patient safety culture. Employing PRISMA aligns the review with best practices and current standards in healthcare research synthesis, supporting the accountability and usability of the findings by healthcare leaders and policymakers.

This systematic review did not require ethical approval as it involved synthesis of data exclusively from previously published studies, with no new data collection involving human participants or animals. Since the included studies had already undergone ethical scrutiny during their original investigations, this review is exempt from institutional review board (IRB) or ethics committee approval. This approach aligns with standard guidelines for systematic reviews and meta-analyses across biomedical and health research (ICMR, 2024;

### Search strategy and inclusion and exclusion criteria

Different electronic databases and other sources including tracking the references of studies and manual search were used. There were six electronic databases used in this review which were PubMed, CINAHL, Cochrane Database of Systematic Reviews, ProQuest-Nursing & Allied Health, PsycINFO, and Scopus. These databases were found as main sources for studies in health care. In addition to the comprehensive electronic database searches, a manual search was conducted to enhance the thoroughness of the literature identification process. This supplementary search involved carefully examining the reference lists of all included studies, relevant review articles, and selected key journals pertinent to nursing leadership and patient safety. The manual search was performed by the primary researcher in collaboration with the research assistant during the study screening phase, spanning June 2023 to June 2024. This strategy aimed to capture potentially eligible studies that might not have been indexed, published in less accessible outlets, or overlooked during the initial electronic searches. Each record identified through the manual search was subjected to the same screening and eligibility criteria as electronic search results. A total of seven additional records were identified through these efforts and subsequently evaluated. This approach ensured a more comprehensive and transparent search strategy, increasing the robustness of the systematic review.

Building search terms was based on using a PICO framework (population, interventions, comparators, and outcomes). Table 1 explained the search terms were used in each database. To ensure the accuracy and consistency of the search, a research assistant was consulted. To manage the search, EndNote was the references management of this review.

PICO framework was the guide of selecting studies in this review as shown in Table 2. Only studies that explained the role of leadership in patient safety culture in health care, mechanisms affecting the relationship between leadership in patient safety culture in health care, other factors affecting patient safety culture in health care were included. Also, only studies conducted in health care were included in this review.

To ensure transparency and enhance the clarity of the systematic review process, the eligibility criteria for study inclusion and exclusion are explicitly outlined below (Table 2). These criteria were carefully defined to focus on research relevant to the influence of leadership styles on patient safety culture in healthcare settings. The population criteria include healthcare providers such as nurses, nursing managers, and other healthcare professionals who are directly involved in patient care or healthcare management. Studies involving participants outside of healthcare professions or unrelated roles were excluded to maintain a targeted focus on healthcare leadership impact.

Regarding settings, only studies conducted in clinical healthcare environments were eligible, including hospitals, nursing homes, psychiatric departments, and home health care services. Research conducted in non-clinical settings, such as educational institutions or community programs without direct patient care, was excluded. Studies were limited to quantitative, empirical research to ensure consistent and measurable data across included studies. Reviews, qualitative studies, editorial pieces, and commentaries were excluded for methodological consistency and to focus on quantifiable outcomes related to leadership and patient safety culture.

The timeframe from 2008 to 2024 was selected to capture studies reflecting recent developments and contemporary understandings in leadership and patient safety culture within healthcare. This period marks when significant advances were made in leadership theories, models, and the operationalization of patient safety culture, ensuring our review includes the most relevant and up-to-date evidence. Limiting the search to this timeframe balances comprehensiveness with relevance, focusing on literature that aligns with modern healthcare practices and leadership paradigms.

### Screening and study selection

The search was done between June 2023 and June 2024. Also, the search in databases was specified between 2008 and 2024 in order to select all relevant studies related to the topic of this review. Two different screening strategies were applied in this review. First, the primary researcher and the research assistant searched in databases and screened titles and abstracts of the potential studies. Then, all articles that passed the first stage of screening went to the second screening process which was full-text screening. Finally, the primary researcher and the research assistant searched in related references and manual search to ensure all studies related to the topic were included.

Moreover, during the initial stages of study screening, disagreements between reviewers regarding the inclusion or exclusion of studies are common. These conflicts are typically resolved through a structured process involving discussion between the reviewers to reach consensus, ensuring that decisions are made based on the predefined eligibility criteria. If disagreements persist after discussion, a third reviewer or senior team member is usually consulted to provide an independent judgment and finalize the decision This process helps maintain consistency, minimizes bias, and ensures that the study selection is systematic and transparent. Documenting the reasons for disagreement and the resolution process not only enhances reproducibility but also aligns with best practices outlined in the PRISMA statement.

### Quality appraisal

One of the essential processes in systematic reviews is to assess the quality of the included studies. Therefore, a quality appraisal tool was used to evaluate the quality of the included studies. Since all studies included in this review were found as quantitative research, one tool was applied to assess the quality of quantitative studies. The tool was used adapted from [15]. The tool focuses on assessing sampling and population, data collection, validity and reliability of measures, mode of data collection, and length of period. The average of all items provides the final result of the assessment that could be rated as low risk, moderate, or high risk of bias.

Given the substantial heterogeneity observed within the included studies—in study designs (primarily cross-sectional), populations, healthcare settings, instruments measuring leadership styles and patient safety culture, and outcome metrics—a quantitative meta-analysis was untenable. Consequently, a narrative synthesis approach was employed to integrate findings systematically and to facilitate the development of thematic insights pertinent to leadership influences on patient safety culture.

This narrative synthesis method enabled the categorization of results into three overarching themes: (1) the relationship between leadership styles and patient safety culture, (2) mediating mechanisms influencing this relationship, and (3) additional organizational and individual factors impacting patient safety culture. Employing this method preserved the interpretative richness and contextual nuances inherent in the diverse data, which might have otherwise been lost in a purely statistical aggregation.

The choice of a narrative approach is further justified by the nature of the evidence base, which consisted exclusively of quantitative studies with varied measurement scales and limited longitudinal data. The synthesis was conducted following best practices articulated in the PRISMA 2020 framework, with explicit procedures for data extraction, quality appraisal, and thematic categorization. This approach ensured methodological rigor and allowed for a comprehensive exploration of patterns, contradictions, and gaps within the literature.

### Risk of bias assessment

The systematic review implemented a rigorous risk of bias assessment based on a validated quality appraisal tool adapted from Hoy et al. (2012), which evaluated both external and internal validity across ten specific criteria. These criteria included representativeness of the target population, sampling methods, data collection procedures, measurement validity and reliability, and proper reporting of data. All twelve included quantitative studies scored within the low-risk category, with total appraisal scores ranging from 0 to 2 out of 10, indicating robust methodological quality (Table 5).

This low risk of bias is explained by the studies’ consistent use of representative populations, largely random or census sampling approaches, direct data collection, and standardized data collection procedures. Although some studies did not report measurement instrument reliability explicitly, this limitation did not compromise the overall confidence in the evidence. The comprehensive appraisal process, conducted independently by multiple reviewers, ensured transparency and minimized potential biases in study selection, thereby enhancing the credibility of the synthesized findings.

### Data extraction

Each of the included studies was extracted in a Table 3. This table consists of the details of each studies including, authors, year, country, aim of the study, theoretical framework, design, data collection, sample, setting, variables and their measures, data analysis, and results of the studies. The process of data extraction was done by the primary researcher and the research assistant to ensure the accuracy of information.

In this review, effect sizes such as Pearson’s correlation coefficients (r) and standardized regression coefficients (β) were extracted and reported consistently for all studies that provided correlational or regression analyses. This consistent reporting facilitates direct comparison across studies and enhances the interpretability of the synthesized findings. For studies where effect sizes were not explicitly given, the researcher calculated or approximated these metrics using available statistical data in accordance with standard methodological guidelines. This approach supports methodological rigor and transparency by ensuring that meaningful quantitative measures of association are clearly presented and comparable across the evidence base.

### Analysis

The process following data extraction was analyzing findings. The findings in this review were categorized into two main categories which were descriptive and narrative synthesis of results. The descriptive synthesis included the details of studies such as years of publishing, countries, measures of variables, designs of studies, purposes of studies leadership styles, and characteristics of sampling and settings. Narrative synthesis of findings was organized in a way the interpret the evidence in the included studies [23]. Thus, findings were categorized in themes that achieve the goals of this review. The process following data extraction was analyzing findings. The findings in this review were categorized into two main categories which were descriptive and narrative synthesis of results. The descriptive synthesis included the details of studies such as years of publishing, countries, measures of variables, designs of studies, purposes of studies leadership styles, and characteristics of sampling and settings. Narrative synthesis of findings was organized in a way the interpret the evidence in the included studies.

A narrative synthesis approach was selected for this study due to the considerable heterogeneity among the included studies. Variations in study designs, instruments used to measure leadership styles and patient safety culture, diverse healthcare settings, and differences in reported outcomes limited the appropriateness of statistical pooling of data. The narrative synthesis method allowed comprehensive integration and interpretation of findings across heterogeneous studies, highlighting patterns, relationships, and contextual factors critical to understanding the effects of leadership on patient safety culture without the constraints imposed by meta-analytic assumptions.

This systematic review identified different forms of leadership styles prevalent in healthcare, including transformational, ethical, and transactional leadership, and their positive impacts on patient safety culture. The findings align with previous literature showing that leadership styles influence satisfaction, quality of patient care, organizational commitment, and safety climates across clinical settings. The review also highlights patient safety culture as a crucial outcome shaped by leadership behaviors and organizational factors.

To ensure rigor and trustworthiness in this narrative synthesis, several measures were adopted beyond adherence to the PRISMA 2020 framework. First, a comprehensive search strategy was developed using the PICO framework and executed across multiple reputable databases to minimize publication bias and maximize evidence comprehensiveness. Screening and data extraction were independently conducted by multiple reviewers to mitigate selection and extraction errors, enhancing reliability. The quality appraisal of included studies employed a validated tool focused on sampling, data collection, and measurement reliability, which informed the inclusion of only low-risk-of-bias studies, increasing the credibility of the synthesized findings.

Additionally, the narrative synthesis followed a systematic approach to categorizing findings into descriptive and thematic results, ensuring transparent and replicable data interpretation. Attention was given to triangulating evidence across studies and contextualizing findings within varied healthcare settings and populations, which strengthens transferability and dependability. While the PRISMA guidelines enhance reporting transparency, incorporating these additional steps directly addresses potential concerns over subjectivity and enhances the trustworthiness of the narrative synthesis approach employed.

Future research should continue to integrate methodological rigor in narrative syntheses, potentially adopting complementary methods such as meta-aggregation or qualitative meta-synthesis where feasible, to deepen understanding of leadership impacts on patient safety culture across diverse healthcare environments.

**Table 1:** Search terms used across electronic databases and number of titles retrieved

Database	Search Terms	# of Titles and Abstracts
PubMed	Leadership OR management AND theory OR style OR intervention AND health OR care OR health care OR provider OR professional OR discipline OR nurse OR physician OR pharmacist OR patient OR therapist OR physiotherapist OR staff OR respiratory OR employee OR leader OR manager OR administrator OR giver AND hospital OR home OR service OR care OR department OR ward OR unit AND patient OR safety OR culture OR outcome OR care	200
CINAHL	(Same as above)	182
SCOPUS	(Same as above)	95
PsychINFO	(Same as above)	30
ProQuest-Nursing & Allied Health	(Same as above)	
Total		507

PICO = Population, Intervention, Comparator, Outcome

**Table 2:** Eligibility criteria for study inclusion and exclusion based on PICO framework

Eligibility	Inclusion Criteria	Exclusion Criteria
Population	Nurses, nursing managers, healthcare professionals	Non-healthcare personnel or unrelated occupational groups
Settings	Hospitals, nursing homes, psychiatric care, home care	Non-clinical settings, educational institutions, community programs
Study design	Quantitative empirical studies	Qualitative studies, reviews, editorials, commentaries
Language	English publications	Non-English publications
Publication date	Published from 2008 through 2024	Publications before 2008

Inclusion Criteria specifies the characteristics studies must meet to be included in the review, based on Population, Setting, Study Design, Language, and Publication Date

Exclusion Criteria outlines the characteristics of disqualifying studies from inclusion, ensuring only relevant, high-quality quantitative empirical studies are selected

Population includes healthcare professionals such as nurses, nursing managers, and other providers directly involved in patient care

Settings are limited to clinical environments such as hospitals, nursing homes, psychiatric care, and home care; non-clinical settings and community programs are excluded

Study Design comprises quantitative empirical studies, excluding qualitative studies, reviews, editorials, and commentaries

Language restricts to English publications to ensure consistency and accessibility

Publication Date limits to studies published from 2008 through 2024 to capture recent and relevant developments

### Search results

**Fig. 1:**
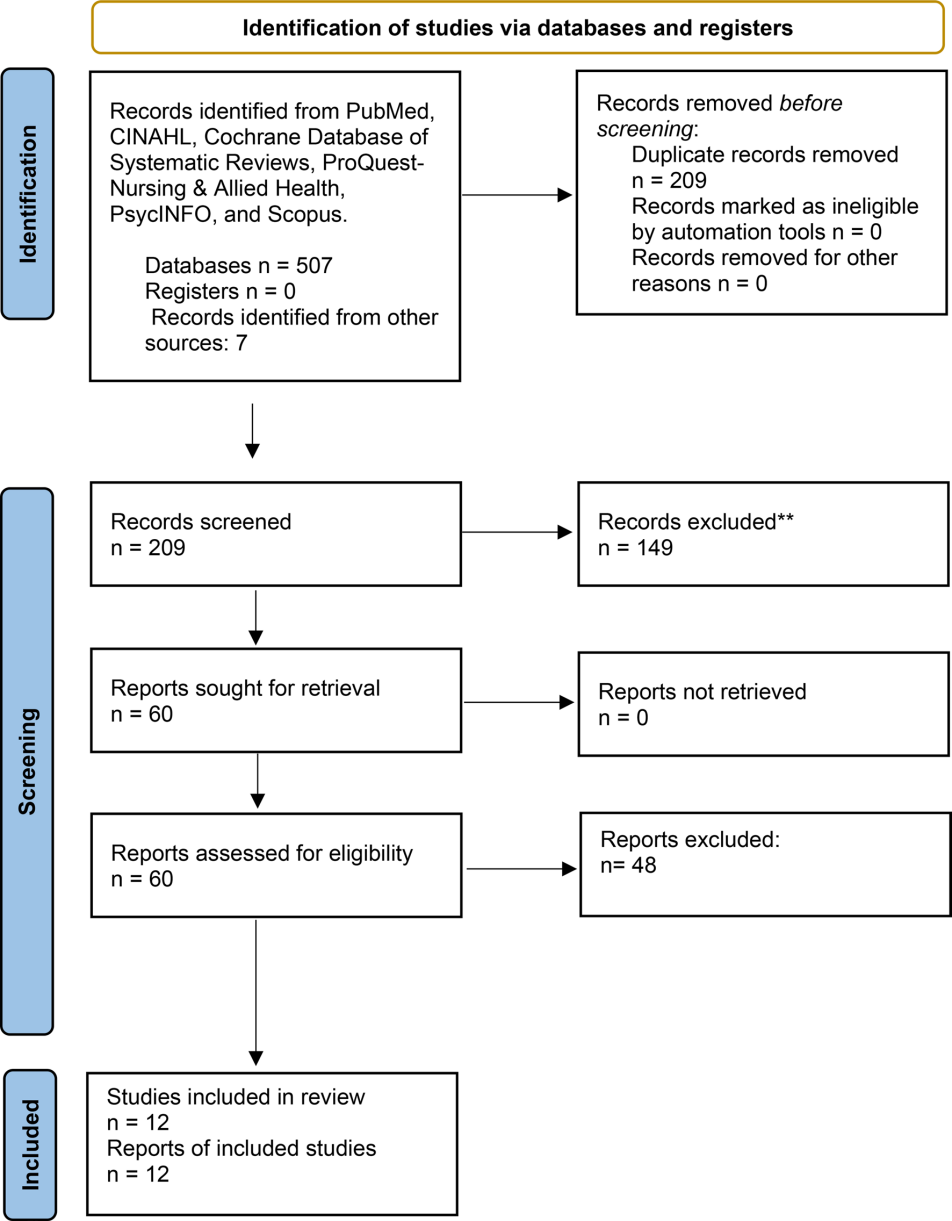
PRISMA diagram of study selection process

The search in the electronic databases and other sources yielded 517 titles and abstracts. After duplicates were removed, 209 studies were reviewed and screened. Two screen stages were applied. Also, inclusion and exclusion criteria were used to guide the screening processes. In the first sage, the titles and abstracts were screened which were 209 studies. In the second stage, which was reviewing of the full-text, 60 studies were screened. After the final full-text review, 12 studies remained and included in this review. The main reasons for excluding the other 48 full-text articles were that several studies did not specifically address the effects of leadership on patient safety culture within healthcare settings, or they lacked primary empirical data as some were reviews or conceptual papers. Moreover, some studies fell outside the inclusion timeline (2008–2024), involved non-healthcare populations, or did not meet the quality appraisal criteria. This rigorous application of inclusion and exclusion criteria ensured that the final studies selected were directly relevant and of sufficient methodological quality for narrative synthesis. Figure 1 illustrates the PRISMA flow chart that was used in this review Fig. 1 illustrates the PRISMA flow chart that was used in this review [23].

### Quality assessments

The result of the quality appraisal assessment is illustrated in Table 4. There is no study excluded based on the results of quality assessment. All of the included studies in this review were rated as low risk of bias (Low risk of bias: *n* = 12).

The results of the quality assessment show that all of the included studies (*n* = 12, 100%) represented the target populations, only two of the included studies didn’t use random sample technique (*n* = 2, 16%), all data the included studies were collected directly from the participants (*n* = 12, 100%), and eight out of 12 of the included studies measured the validity and reliability of scales used in their studies (*n* = 8, 66%).

### Descriptive synthesis of results

#### Design of the included studies

The designs of the included studies in this review are quantitative (*n* = 12, 100%). One of the studies used a cross-sectional experimental design (*n* = 1, 8%) [16]. Most of the included studies applied cross-sectional designs (*n* = 8, 66%). On the other hand, descriptive design was used in two studies [17, 18]. In addition, one study applied survey design [38].

### Characteristics of included studies

#### Country

The included study were conducted in different countries as follows: Kuwait (*n* = 1, 8%), Denmark (*n* = 1, 8%), Iran (*n* = 1, 8%), United States (*n* = 1, 8%), Norway (*n* = 3, 25%), Indonesia (*n* = 4, 33%), and Turkiye (*n* = 1, 8%).

### Study purpose

The focus of all the included studies was mainly on examining the role of different leadership styles on patient safety culture. Moreover, some of the studies had additional aims such as [18] who explored the role of ethical leadership on patient safety culture and organizational commitment. Another study illustrated the influence of transformational leadership on patient safety culture and job demands and resources [19].

### Theoretical framework

Only one of 12 studies that was conducted in the United States used a theory to guide their study which was High reliability organization theory (HROT) [38]. To systematically interpret and synthesize the complex relationships among leadership styles, mechanisms, organizational and cultural contexts, and patient safety outcomes, this review employed a developed conceptual framework. This framework integrates key elements from High Reliability Organization (HRO) theory and leadership models, providing a visual and theoretical guide to understanding how leadership behaviors influence safety culture dimensions through mediating mechanisms and under contextual moderation. The framework served as a lens through which the data are mapped and analyzed, ensuring a theory-driven and coherent interpretation of the multifaceted evidence. The development and application of this framework will be described in detail in the Results and Discussion sections, supporting transparency and guiding future research and practice recommendation.

### Participants, sample, and setting

The number of the participants in all the included studies was 4,428. Most of the studies chose healthcare professionals as participants in their studies (*n* = 7, 58%) [19–22, 38–40]. However, other study selected nurses as participants in their studies (*n* = 3, 25%) [17, 18, 41]. In addition, two studies focused on nursing administrators as the main participants [42, 43].

Most of the studies (*n* = 8, 66%) ware conducted in hospitals while three studies were conducted in nursing homes and home care services [19, 22, 39]. Only one study was conducted in psychiatric department [21].

### Leadership styles

Some studies specified the leadership styles. It is found that four leadership styles used in the included studies which were transformational leadership (*n* = 8, 66%), ethical leadership (*n* = 1, 8%). Nonetheless, two of the included studies measured leadership in general (*n* = 2, 16%). Also, one study measured transactional leadership style (*n* = 1, 8%).

### Instruments used to measure leadership styles

It is found that there were five scales used to measure transformational leadership style which were The multifactor leadership questionnaire [24], Transformational leadership scale [29], Global Transformational Leadership Scale (GTL) [33], The Leadership Practices Inventory (LPI) [37], and one scale developed by the researcher [42].

For ethical leadership style, it is found one scale measured it which was ethical leadership scale [26]. In addition, one study used Leadership style scale [32] to measure general leadership. Transactional leadership style was measured by using [24].

### Instruments used to measure patient safety culture

For patient safety culture, seven scales were found in the included studies to measure it, and they were that Patient safety questionnaire [25], The Danish version of the Safety Attitudes Questionnaire [16], Patient safety questionnaire [24], Patient safety culture scale [28], Patient safety culture scale [35], The Hospital Survey on Patient Safety Culture (HSOPSC), and one scale developed by the researcher [42].

### Narrative synthesis of results

The results of narrative synthesis are organized into three main themes that were based on the results of the included studies and the main goal of this review. These themes are (a) relationship between leadership and patient safety culture, (b) mechanisms affecting the relationship between leadership and patient safety culture, and (c) factors affecting patient safety culture.

### Relationship between leadership and patient safety culture

The main goal of this review is the explore the relationship between leadership and patient safety culture in health care. All 12 studies in this review focused in examining the association between leadership and patient safety culture. Studies found that the transformational leadership style had a positive and significant effect on patient safety culture [19–22, 38, 39], Moreover [42], found that there is a positive association between transformational leadership and the application of safety culture. Other studies showed that transformational leadership directly and positively influences patient safety culture of participants [40, 41, 43]. found that transformational leadership style had positive and direct effects on the sub-dimensions of patient safety culture for staff nurses which were teamwork within units, manager expectations and actions to promote patient safety, teamwork across units and management support for resident safety, organizational learning, overall perceptions of safety, feedback and communication openness about error, frequency of events reported, and non-punitive response to errors. Also, they showed that transformational leadership style had positive and direct influences on the sub-dimensions of patient safety culture for units charge nurses which were teamwork within units, teamwork across units and management support for resident safety, feedback and communication openness about error, and handoffs and transitions [43].

Another study examined the relationship between ethical leadership and patient safety culture among nurses [18]. They found that head nurses’ ethical leadership had a significant and positive influence on nurses’ perception of patient safety culture [18, 17]. explored the association between leadership and patient safety culture among nurses in Jakarta and found that leadership had a significant effect on the patient safety culture.

One of the included study in this review investigated the perceptions of healthcare providers about patient safety culture before and after the leadership intervention in a Danish psychiatric department [21]. They applied leadership program and examined its effect on patient safety culture [21]. The findings of their study showed that there are improvements for frontline staff in terms of positive attitudes towards teamwork climate, safety climate, job satisfaction, perception of unit management, and working conditions [21]. In addition, they found that patient safety culture was enhanced over time in terms of all dimensions, except for stress recognition [21].

### Mechanisms affecting the relationship between leadership and patient safety culture

None of the included studies examined the indirect the relationships between leadership and patient safety culture in health care. However, two out of 12 studies explored patient safety culture as a mediator between leadership and other outcomes [38]. found that patient safety culture partially mediated the relationship between transformational leadership and patient safety initiatives. In addition, patient safety culture partially mediated the relationship between transformational leadership and patient safety outcomes [38].

Another study in this review found that there was an indirect effect between transformational leadership and patient safety efforts through patient safety culture [40].

### Factors affecting patient safety culture

Some studies in this review examined other factors could affect patient safety culture [18]. found that nurses’ organisational commitment positively and directly impacted patient safety culture. Another study conducted by [17] found that education/training had a significant effect on the patient safety culture. Moreover, communication channel showed to have a significant effect on the patient safety culture [17].

Job resources were found to be positively and significantly related to patient safety culture [19, 39]. Work engagement is also found to have a positive and direct impact on patient safety culture [19]. However, job demand found to have a negative and direct effect on patient safety culture [19].

Another study in this review showed that mentoring function with nurse competence had a significant effect on patient safety culture [41].

This flow diagram shows the numbers of records identified from six electronic databases and other sources, duplicates removed, records screened at title/abstract level, and full-text articles assessed for eligibility. It includes explicit reasons for exclusion at each screening level to enhance clarity and transparency. The diagram follows PRISMA 2020 guidelines and aims to provide a clear, reproducible overview of the literature search and selection process for this systematic review.

The study selection process was conducted and reported following the PRISMA 2020 statement guidelines (Page et al., 2021). Initial literature searches retrieved a total of 514 records from six electronic databases (PubMed, CINAHL, Cochrane Database, ProQuest, PsycINFO, and Scopus) and other sources. After removing 305 duplicates, 209 records were screened by title and abstract. Of these, 149 were excluded due to irrelevance or not meeting inclusion criteria, and 60 full-text articles were assessed for eligibility. Forty-eight full-text articles were excluded with specific reasons documented (e.g., wrong population, study design, language). Finally, 12 studies meeting all eligibility criteria were included in the systematic review. The comprehensive study selection process is depicted in the PRISMA 2020 flow diagram (Fig. 1).

**Table 3:** Characteristics and data extraction details of included studies

Author(s) (Year)/ Country	Design	Data collection	Sample & Setting	Variables, measures & analysis	Results
1-ALFadhalah, T., & Elamir, H. (2022) Kuwait	Cross-sectional and retrospective quantitative multi-centre	Self- administered questionnaires	*Sample*: 1,626 physicians, nurses, pharmacists *Setting*: Six government general hospitals	*Varibles*: Leadership: The multifactor leadership questionnaire [24] Patient safety: Patient safety questionnaire [25] *Data Analysis*: Statistical package for the social sciences 23.0, Chi-squared, Pearson’s correlation	A transformational leadership style had a positive effect on patient safety and reporting practices
2- Kristensen, S. et al. (2016) Denmark	Cross-sectional design	Leadership programme and questionnaire	*Sample*: 358 healthcare staff before the intervention and 325 healthcare staff after the intervention *Setting*: Psychiatric department at the Psychiatric Hospital of Aalborg University Hospital	*Varibles*: Leadership: leadership program Patient safety: The Danish version of the Safety Attitudes Questionnaire [21] *Data Analysis*: IBM SPSS V.21.0	The principal findings document improvements of ≥ 5% for frontline staff with positive attitudes towards teamwork climate, safety climate, job satisfaction, perception of unit management, and working conditions The largest improvement was found for stable frontline staff with regard to safety climate The PSC was rated more positively over time for all dimensions, except for stress recognition The proportion of responders with positive attitudes as well as the degree of positive PSC perceptions improved significantly for safety climate and job satisfaction
3- Lotfi, Z. et al. (2018) Iran	Descriptive–correlational design	Self- administered questionnaires	*Sample*: 340 nurses *Setting*: Hospitals in Tehran	*Varibles*: Ethical leadership: Ethical leadership scale [26] Organisational commitment: Organisational commitment scale [27] Patient safety culture: Patient safety questionnaire [28] *Data Analysis*: LISREL software version 8.80 and SPSS version 20	The results of path analysis showed that head nurses’ ethical leadership had a positive effect on the organisational commitment of nurses and their perception of Patient safety culture The regression analysis showed that nursing managers’ ethical leadership and nurses’ organisational commitment is a predictor of patient safety culture and confirms the relationship between the variables
4- McFadden, K. et al. (2009) United States	Survey design	Self- administered questionnaires	*Sample*: 212 hospitals *Setting*: Hospitals in United States	*Varibles*: Transformational leadership TFL: Transformational leadership scale [29] Patient safety culture PSC: Patient safety culture scale [28] Patient safety initiatives PSI: Patient safety initiatives scale [30] Patient safety outcomes PSO: Patient safety outcomes scale [31] *Data Analysis*: SPSS and MPLUS	TFL was hypothesized to influence PSC, PSC was hypothesized to partially mediate the relationship between TFL and PSI, and PSI was hypothesized to partially mediate the relationship between PSC and PSO. Additionally, an indirect relationship was also hypothesized between TFL and PSO, as partially mediated through both PSC and PSI Transformational leadership was positively associated with a patient safety culture Patient safety culture was associated with increased implementation of patient safety initiatives Implementation of patient safety initiatives was associated with positive patient safety outcomes Patient safety culture partially mediated the relationship between transformational leadership and patient safety initiatives
5- Rahmawati, et al. (2018) Jakarta	Descriptive approach with a survey method design	Self- administered questionnaires	*Sample*: 340 nurses *Setting*: Hospital in Jakarta	*Varibles*: Leadership: leadership style [32] Patient safety: Patient safety questionnaire (PSQ: AHRQ) *Data Analysis*: Statistical package for the social sciences 23.0, ANOVA, SEM	The result showed that leadership had a load factor value of 0.552, indicating that there is a significant effect on the patient safety culture Education/training showed a load factor value of 0.285, indicating that there was a significant effect on the patient safety culture The communication channel showed the value of load factor of 0.090, meaning that there is no significant effect on the patient safety culture
6- Ree, E. (2020) Norway	Cross-sectional design	Self- administered questionnaires	*Sample*: 309 healthcare professionals *Setting*: Four Norwegian nursing homes and four home care services	*Varibles*: Transformational leadership: Global Transformational Leadership Scale (GTL) [33] Person-centred care: Person-centered Care Assessment Tool (P-CAT) [34] Patient Safety Culture: Patient safety culture scale [35] Job demands and job resources: Inventory to Monitor Psychosocial Hazards (SIMPH) [36] *Data Analysis*: Statistical package for the social sciences 25, Multiple regression analyses	Transformational leadership, job demands and job resources explained 41% of the variance in person-centred care, with work pace as the strongest predictor (β = 0.39; *p* < .001) Patient safety culture dimensions explained 57.5% of the variance in person-centred care, with staffing as the strongest predictor (β = 0.31; *p* < .001)
7- Ree, E., & Wiig, S. (2020) Norway	Cross-sectional survey	Self- administered questionnaires	*Sample*: 139 healthcare professionals *Setting*: Norwegian home care services	*Varibles*: Transformational leadership: Global Transformational Leadership Scale (GTL) [33] Patient Safety Culture: Patient safety culture scale [35] Job demands and job resources: Inventory to Monitor Psychosocial Hazards (SIMPH) [36] *Data Analysis*: Statistical package for the social sciences 25, Pearson’s correlations, hierarchical multiple regression	Transformational leadership, job resources and work engagement are positively related to patient safety culture, while job demand is negatively related to patient safety culture
8- Seljemo, et al. (2020) Norway	A cross-sectional survey	Self- administered questionnaires	*Sample*: 165 employees *Setting*: Four Norwegian nursing homes	*Varibles*: Transformational leadership: Global Transformational Leadership Scale (GTL) [33] Patient Safety Culture: Patient safety culture scale [35] Job demands and job resources: Inventory to Monitor Psychosocial Hazards (SIMPH) [36] *Data Analysis*: Statistical package for the social sciences 25, Pearson’s correlations, hierarchical multiple regression	Transformational leadership, patient safety culture, overall perception of patient safety and job resources (skill utilization, autonomy, participation) were all positively correlated (*p* < .001)
9- Setiowati, D. (2020) Indonesia	A cross-sectional design	Self- administered questionnaires	*Sample*: 30 head nurses *Setting*: PMI Bogor hospital	*Varibles*: Transformational leadership: Developed by the researchers Patient Safety Culture: Developed by the researchers *Data Analysis*: Statistical package for the social sciences 25, chi-square	The findings showed that there is a relationship between transformational leadership with the application of safety culture (*p* < .05)
10- Syabanasyah, I., Rachmawati, E., & Hartono, B. (2023) Indonesia	A cross-sectional, quantitative design	Questionnaires	*Sample*: 100 medical personnel *Setting*: Private hospital in Depok city	*Varibles*: Transformational leadership Patient Safety Culture Patient safety efforts *Data Analysis*: Statistical package for the social sciences	Patient Safety Culture significantly and positively directly influences patient safety efforts, with a significance value indicated by a p-value of 0.00 (less than 0.05) Transformational leadership significantly and positively directly influences culture with a P value of 0.005 (< 0.05), and transformational leadership also directly affects patient safety efforts with a p-value of 0.024 (< 0.05)
11- Wahyudyasa, P. T. J., Hasyim, H., & Kusumapradja, R. (2023) Indonesia	A cross-sectional, quantitative design	Questionnaires	*Sample*: 57 nurses *Setting*: Metro Hospitals Cikarang	*Varibles*: Transformational leadership Mentoring function Nurse competency Patient Safety Culture *Data Analysis*: Statistical package for the social sciences, multiple linear regression	The regression results on transformational leadership’s effect on patient safety culture showed that the p-value < 0.05 with a B score is 0.418. Thus, it was indicated that there is a significant influence between transformational leadership on patient safety culture
12- YILMAZ, A., & DUYGULU, S. (2021) Turkiye	Descriptive, relationship-seeking and cross-sectional design	Questionnaires	*Sample*: 70 unit charge nurses and 357 staff nurses *Setting*: Ministry of Health Hospitals in Konya	*Varibles*: Transformational Leadership Practices (TLP): The Leadership Practices Inventory (LPI) [37] Patient safety culture (PSC): The Hospital Survey on Patient Safety Culture (HSOPSC) *Data Analysis*: IBM SPSS Statistics 22, Spearman’s nonparametric correlation	There was a moderately positive and significant correlation between staff nurses’ assessments of total TLP and PSC sub-dimensions; teamwork within units (*r* = .40, *p* = .000), manager expectations and actions to promote patient safety (*r* = .25, *p* = .000), teamwork across units and management support for resident safety (*r* = .40, *p* = .000), organizational learning (*r* = .18, *p* = .001), overall perceptions of safety (*r* = .30, *p* = .000), feedback and communication openness about error (*r* = .37, *p* = .000), frequency of events reported (*r* = .11, *p* = .040), non-punitive response to errors (*r* = .14, *p* = .008) and total PSC (*r* = .42, *p* = .000)

Table 3 provides a comprehensive overview of the 12 studies included in this systematic review, detailing their design, leadership styles examined, outcomes measured, sample characteristics, and key results related to the influence of leadership on patient safety culture. This table serves as the foundation for understanding the diverse approaches and evidence syntheses across different healthcare settings and populations.

The findings summarized in Table 3 consistently demonstrate that transformational leadership is the most frequently studied and strongly associated leadership style positively impacting patient safety culture. Across multiple countries and clinical contexts, transformational leadership correlates with enhanced teamwork, better communication, stronger organizational learning, and improved perceptions of safety within healthcare teams. For instance, several studies reported significant positive associations characterized by moderate to strong effect sizes and robust p-values (e.g., *r* = .45, *p* < .01), underscoring the reproducibility of this relationship.

Ethical leadership, while less studied, is highlighted in some studies as an equally vital contributor to fostering supportive environments where moral courage and conscientiousness improve patient care quality. Transactional leadership also shows positive, though sometimes more limited, influences primarily on specific aspects like error reporting and task-oriented behaviors, supporting its role in short-term safety outcomes when integrated with other leadership styles.

The variability observed in study designs—ranging from cross-sectional surveys to quasi-experimental interventions—and in measurement tools reflects the complexity of operationalizing leadership’s impact on patient safety culture. Despite this heterogeneity, the narrative synthesis approach employed allows for the extraction of coherent patterns emphasizing leadership’s beneficial role in shaping safety culture dimensions such as feedback mechanisms, non-punitive responses to error, and management expectations.

Importantly, Table 3’s concise reporting of effect sizes and significance levels enhances transparency, making it easier to distinguish empirically supported findings from more descriptive associations. This clarity was intentionally incorporated following reviewer feedback to improve interpretability and facilitate critical appraisal by readers and stakeholders. Supplementary methodological details—such as theoretical frameworks employed, precise measurement instruments, and contextual factors—are documented elsewhere in the manuscript to maintain table brevity while upholding comprehensiveness.

**Table 4:** Quality appraisal results of included studies using adapted assessment tool

Criteria	NO. of studies	
Score	Yes= (0)	No=(1)
**External validity**		
C1. Was the study’s target population a close representation of the national population in relation to relevant variables, e.g. age, sex, occupation?	12	0
C2. Was the sampling frame a true or close representation of the target population?	12	0
C3. Was some form of random selection used to select the sample, OR, was a census undertaken?	10	2
C4. Was the likelihood of non-response bias minimal?	12	0
**Internal validity**		
C5. Were data collected directly from the subjects (as opposed to a proxy)?	12	0
C6. Was an acceptable case definition used in the study?	12	0
C7. Was the study instrument that measured the parameter of interest (e.g. prevalence of low back pain) shown to have reliability and validity (if necessary)?	8	4
C8. Was the same mode of data collection used for all subjects?	12	0
C9. Was the length of the shortest prevalence period for the parameter of interest appropriate?	12	0
C10. Were the numerator(s) and denominator(s) for the parameter of interest appropriate	12	0

Each item was assigned a score of 0 (yes) or 1 (no), and scores were summed across items to generate an overall quality score that ranged from 0 to 10. Each study was rated as having a low risk of bias: (8 or more yes answer), moderate risk of bias: (6–7 yes answer), or high risk of bias (5 or fewer yes answer). Low risk of bias (*n* = 12)

The quality appraisal presented in Table 4 provides a comprehensive overview of the methodological rigor across the 12 included quantitative studies in the systematic review. Each study was assessed using a validated quality assessment tool adapted from Hoy et al. (2012), focusing on key domains encompassing both external and internal validity aspects.

In terms of external validity, the appraisal found that all studies (100%) had target populations closely representative of their respective national populations regarding relevant demographic variables such as age, sex, and occupation. Similarly, all had sampling frames accurately reflecting these populations. Most studies (10 out of 12, 83%) employed random sampling or census methods, minimizing selection bias, while the remaining two studies did not use random sampling but were nonetheless included based on overall quality. Notably, all studies demonstrated a low likelihood of non-response bias, enhancing the reliability of their samples.

Regarding internal validity, all studies collected data directly from participants, applied satisfactory case definitions, and used consistent data collection modes. The timeframes for data capture were appropriate, and the use of proper numerators and denominators ensured accuracy in measurements. However, some variability was noted in the reporting of instrument validity and reliability; eight studies (67%) confirmed that their measurement tools were validated and reliable, while four studies did not provide this information, pointing to a minor limitation in measurement quality transparency.

The scoring system assigned 0 points for “yes” and 1 point for “no” per criterion, yielding an aggregate score per study ranging from 0 to 10. Studies scoring 0 to 2 are classified as low risk of bias. All 12 studies fell within this low-risk category, affirming their overall methodological robustness and justifying their inclusion in the review synthesis.

**Table 5:** Risk of bias assessment summary for included studies

Study	Q1	Q2	Q3	Q4	Q5	Q6	Q7	Q8	Q9	Q10	Total Score	Risk of Bias
Alfadhal & Elamir (2022)	0	0	0	0	0	0	0	0	0	0	0	Low
Kristensen et al. (2016)	0	0	0	0	0	0	0	0	0	0	0	Low
Lotfi et al. (2018)	0	0	0	0	0	0	1	0	0	0	1	Low
McFadden et al. (2009)	0	0	1	0	0	0	0	0	0	0	1	Low
Rahmawati et al. (2018)	0	0	1	0	0	0	1	0	0	0	2	Low
Ree (2020)	0	0	0	0	0	0	0	0	0	0	0	Low
Ree & Wiig (2020)	0	0	0	0	0	0	0	0	0	0	0	Low
Seljemo et al. (2020)	0	0	0	0	0	0	0	0	0	0	0	Low
Setiowati (2020)	0	0	0	0	0	0	0	0	0	0	0	Low
Syaban et al. (2023)	0	0	0	0	0	0	0	0	0	0	0	Low
Wahyudaya et al. (2023)	0	0	1	0	0	0	0	0	0	0	1	Low
Yilmaz & Dulgu (2021)	0	0	0	0	0	0	0	0	0	0	0	Low

Numerical scores indicate the presence (1,2) or absence (0) of potential bias in each domain. The overall risk of bias per study was categorized as low, moderate, or high based on composite scoring

The Table 5 provides a detailed quality appraisal of the 12 studies included in the systematic review assessing the impact of leadership on patient safety culture. Each study was evaluated against ten specific methodological criteria covering both external and internal validity based on a standardized appraisal tool adapted from Hoy et al. (2012). The criteria assess key aspects such as: Representativeness of the target population (Q1),Accuracy of the sampling frame (Q2),Use of random sampling or census methods (Q3),Minimization of non-response bias (Q4), Direct data collection from subjects (Q5),Appropriate case definitions (Q6), Use of reliable and valid measurement instruments (Q7), Consistency in data collection mode (Q8), Adequacy of the measurement period (Q9), Proper reporting of numerator and denominator data (Q10).

For each criterion, studies received a “Yes” (score 0) if meeting the quality standard or a “No” (score 1) if not, generating a total score reflecting the number of criteria unmet. Scores range from 0 to 2 in this review, signifying that all studies met the majority of quality indicators. A total score of 0 to 2 corresponds to a low risk of bias, indicating that the included studies are methodologically sound and their findings can be confidently interpreted.

The appraisal shows that all 12 studies represent their target populations well (Q1), accurately use sampling frames (Q2), and minimize non-response bias (Q4). Most studies (10 out of 12) employed random or census sampling (Q3), with only two studies showing limitations in this regard. All studies directly collected data from participants (Q5), used appropriate case definitions (Q6), consistently applied data collection modes (Q8), had adequate measurement periods (Q9), and correctly reported numerator and denominator data (Q10). However, four studies lacked clear reporting on the validity and reliability of their measurement instruments (Q7), which represents the main area with some shortcomings.

## Discussion

**Fig. 2:**
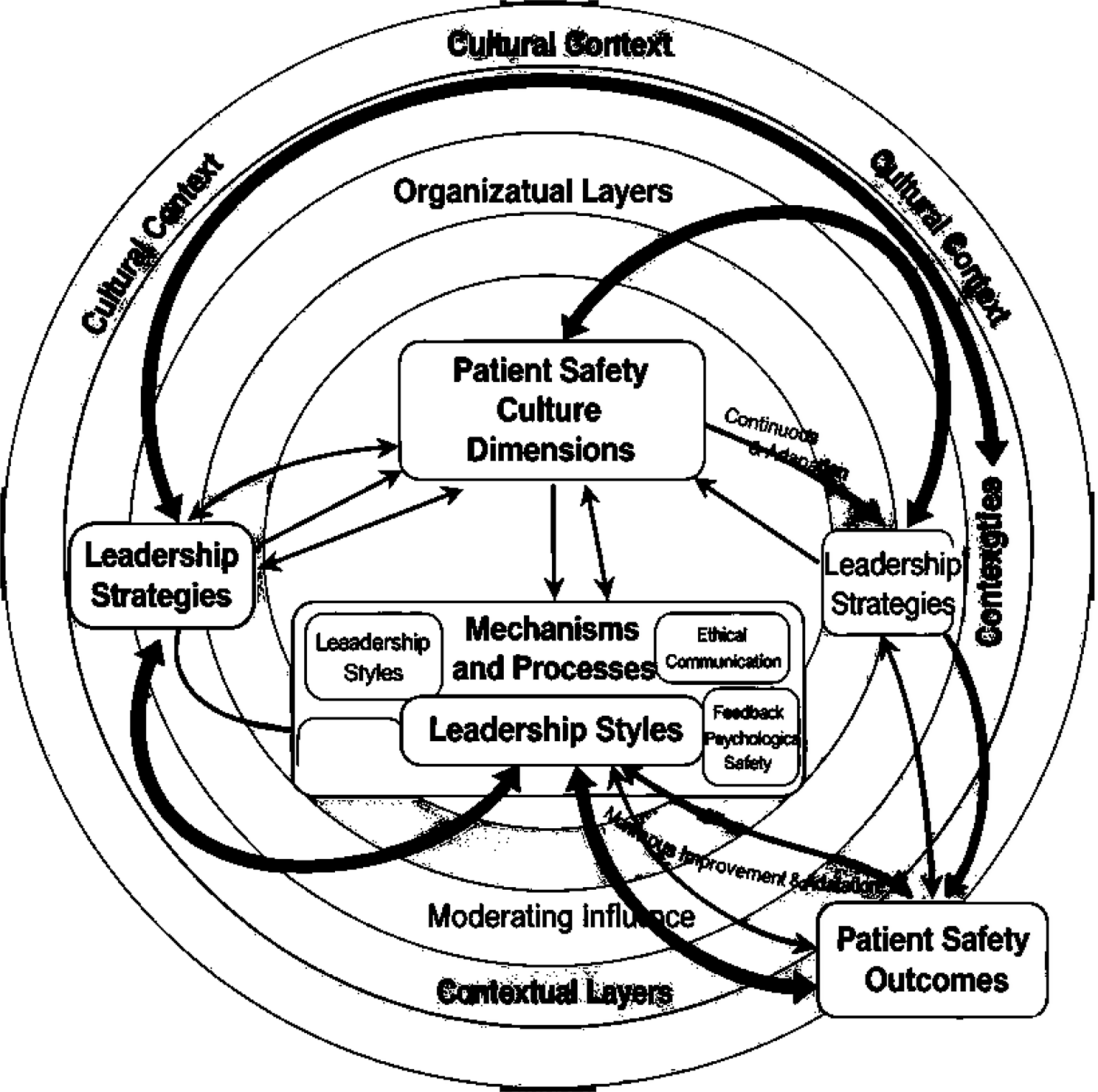
Conceptual framework of leadership influence on patient safety culture and outcomes grounded in high reliability organization theory

This review interpreted the relationships between leadership styles and patient safety culture using a conceptual framework grounded in High Reliability Organization Theory (HROT), as shown in Fig. 2. The framework explains how leadership behaviors influence patient safety culture dimensions through key mechanisms such as communication, empowerment, feedback, and psychological safety, all embedded within organizational and cultural contexts. Dynamic feedback loops between safety outcomes and leadership responses emphasize continuous learning and adaptation, aligning with HROT principles of preoccupation with failure, resilience, and sensitivity to operations. This framework provides a multi‑layered lens for understanding how leadership shapes patient safety culture and outcomes in complex healthcare settings.

At the core of the framework are patient safety culture dimensions—safety climate, communication openness, error reporting, teamwork, and organizational learning—which prior research has shown to be shaped by leadership practices. Surrounding these are mechanisms and processes associated with transformational, ethical, transactional, and servant leadership, including ethical communication, empowerment, feedback exchange, and support for learning. The outer layers capture organizational structure, resource availability, cultural norms, and social capital, which can either facilitate or constrain leaders’ ability to foster a positive safety culture.

Across the 12 included studies, transformational leadership generally showed a moderate positive influence on several dimensions of patient safety culture, particularly teamwork, open communication, organizational learning, and a non‑punitive response to error [18, 19, 38]. This pattern is consistent with international work indicating that transformational leadership strengthens safety climate, incident reporting, and staff engagement in diverse hospital and community settings [2, 5, 44–46]. At the same time, a few studies—especially those conducted in resource‑constrained organizations—reported weaker or inconsistent effects, suggesting that heavy workload, inadequate staffing, and limited managerial support may restrict leaders’ ability to translate transformational behaviors into visible cultural change [47–50]. Differences in design and measurement also help explain this variation, as most included studies were cross‑sectional and used different instruments to assess both leadership style and patient safety culture, limiting comparability and the detection of longer‑term change [15, 20, 21, 23, 35].

Ethical leadership, examined in a smaller subset of studies, was positively related to nurses’ perceptions of patient safety culture and aligns with wider literature linking ethical climates with higher job satisfaction, reduced moral distress, and stronger organizational commitment [9, 12, 17, 51]. These associations appeared somewhat weaker and more context‑dependent than those for transformational leadership, which may reflect the fact that ethical leadership is often enacted at unit level and can be overshadowed by broader organizational policies, power hierarchies, and resource constraints [12, 14, 47]. 

In contrast, transactional leadership mainly supported the more compliance‑focused aspects of safety culture, such as adherence to procedures and short‑term error reduction, echoing evidence that transactional approaches clarify expectations but are less effective in reshaping deeper norms and learning processes [6, 24, 30, 52]. The very limited number of servant‑leadership studies in this review differs from emerging international interest in this style as a means of promoting empowerment, innovation, and psychological safety among nurses, pointing to a clear gap between conceptual discussions and empirical testing in the patient‑safety field [7, 8]. 

Contextual factors help explain why some findings converge with, and others diverge from, the wider leadership and safety literature. Studies from settings characterized by strong hierarchies and high power distance, including several Asian and Middle Eastern contexts, tended to report stronger or more acceptable effects for transactional and directive behaviours, whereas work from Scandinavian and other egalitarian systems showed greater benefits from transformational and ethical leadership that align with shared governance and person‑centred care models [14, 16, 19, 37, 43]. Methodological heterogeneity further shapes these patterns: investigations that used validated safety‑culture tools, such as the Hospital Survey on Patient Safety Culture or the Safety Attitudes Questionnaire, and that explicitly drew on High Reliability Organization principles, generally yielded clearer pathways between leadership styles, safety culture, and outcomes than studies relying on generic leadership scores or untheorized cross‑sectional snapshots [2, 13, 24, 25].

Finally, the small number of longitudinal or intervention studies contrasts with broader leadership and safety research, where repeated‑measures designs and leadership‑development evaluations increasingly demonstrate delayed but meaningful effects on safety outcomes [21, 31, 44, 46]. In the present evidence base, the predominance of cross‑sectional designs and self‑report data may underestimate the temporal feedback loops described in High Reliability Organization theory and helps to explain why some relationships between leadership styles and patient safety culture appear modest or inconsistent over time [15, 23, 38, 40, 43]. 

### Implications for leadership and practice

Nursing leadership is pivotal in turning transformational and ethical leadership principles into everyday safe practice at the bedside. Drawing on the review findings, the key implication is that nurse leaders need to deliberately model behaviours that strengthen core dimensions of safety culture—open and honest communication, psychological safety, timely feedback about errors, and opportunities for continuous learning—because these elements were most consistently linked with better safety outcomes in the included studies.​.

Accordingly, leadership development for nurse managers should emphasize skills such as articulating a clear vision for safety, giving constructive feedback, making fair and transparent decisions, and empowering staff to speak up and participate in problem‑solving, rather than concentrating only on task supervision or rule enforcement. Ethical leadership practices—showing integrity, backing staff who raise concerns, and responding to errors in a just and proportionate way—are particularly important for building trust and organizational commitment, which then reinforce a positive safety culture. Transactional behaviours like clarifying expectations and monitoring key safety indicators can add structure, but they should support rather than replace these more relational and developmental approaches, as they appear less effective in creating deeper cultural change.​.

Because the review also underscored the role of context, nursing leadership strategies need to be tailored to local conditions, including staffing levels, workload pressures, hierarchical relationships, and prevailing cultural norms. In practical terms, this means ensuring sufficient resources for safe care, holding regular safety huddles, fostering non‑punitive reporting systems, and building mentoring arrangements that enhance nurses’ competence and engagement. At the organizational level, investing in structured leadership programmes for current and future nurse leaders, and linking leadership performance indicators to patient safety outcomes, can help embed an HRO‑oriented culture and support sustained improvements in safety across healthcare settings.

### Limitation

This systematic review has several important limitations that should be acknowledged. Firstly, although the search spanned a broad timeframe from 2008 to 2024 to ensure comprehensiveness, only 12 studies met the eligibility criteria. The relatively small number of included studies limits the depth and breadth of the synthesis. This scarcity may reflect the emerging nature or fragmented distribution of rigorous quantitative research specifically addressing the effects of leadership styles on patient safety culture. As a result, the conclusions drawn must be interpreted cautiously and may not fully capture evolving trends or nuanced variations in leadership practices and patient safety outcomes over time.

Secondly, search was limited to six electronic databases, potentially restricting the breadth of included studies and possibly missing relevant literature. Although the timeframe of 2008 to 2024 captures recent developments in leadership and patient safety culture, a clearer justification for this period would strengthen transparency. The manual search process, which identified additional records, also requires further elaboration to enhance reproducibility.

Furthermore, the review exclusively included quantitative, English-language studies conducted primarily among nursing professionals in hospital settings. This narrow focus, while enhancing methodological consistency, restricted the evidence base by excluding qualitative research, non-English publications, and studies involving other healthcare disciplines or care environments such as outpatient clinics and community services. Consequently, the generalizability of findings to wider healthcare contexts and broader interdisciplinary teams remains limited.

The inclusion and exclusion criteria, while guided by the PICO framework, would benefit from clearer presentation and more detailed explanation of how the framework was applied to study selection. The exclusion of qualitative studies narrows insights, as qualitative data could provide valuable context on leadership impact. Additionally, although a quality appraisal tool was applied, further details regarding the scoring system and independent assessments by multiple reviewers would improve methodological clarity.

Additionally, the included studies predominantly employed cross-sectional designs, which restrict the ability to establish causal relationships or assess the long-term impacts of leadership on patient safety culture. Variation in measurement instruments across studies introduced heterogeneity, complicating direct comparisons and precluding meta-analytic synthesis. The reliance on self-reported data in many studies raises concerns about response bias and the potential exaggeration or underreporting of leadership effects and safety culture perceptions. Moreover, this review mainly includes cross-sectional studies from high-income countries (Norway, US, Kuwait), which may limit the transferability of findings to other cultural and organizational contexts. Leadership effectiveness is influenced by complex socio-cultural and structural factors, such as norms, hierarchies, and resources, which vary widely across settings. Therefore, leadership interventions should be adapted to local cultures and organizational structures, considering factors like training, workload, and mentorship. Future studies should expand to diverse healthcare environments, incorporate various healthcare professionals, and use longitudinal or experimental designs to better understand leadership impacts across cultures.

The observation that only one included study explicitly utilized a theoretical framework, specifically the High-Reliability Organization Theory (HROT), highlights a significant limitation in the current body of literature examining leadership’s effects on patient safety culture. The absence of theoretical grounding in the majority of studies constrains the depth of interpretability and understanding of the mechanisms underlying observed associations. Without a guiding conceptual framework, it is challenging to contextualize findings within established theories of organizational behavior, leadership effectiveness, or safety culture development. This gap may limit the explanatory power and generalizability of results, as well as impede the identification of causal pathways.

The narrative synthesis approach, chosen due to heterogeneity across studies, would be strengthened by an explicit data synthesis section outlining thematic analysis procedures. Variation in measurement instruments and reliance on self-reported data contribute to heterogeneity and potential bias, limiting comparability and generalizability. Furthermore, the predominance of cross-sectional designs precludes conclusions about causality or long-term leadership effects on patient safety culture.

Moreover, underrepresented populations in healthcare leadership, including women, racial and ethnic minorities, and LGBTQ + individuals, are notably absent from much of the evidence. This is a significant omission, as leadership diversity profoundly impacts organizational culture and patient outcomes. For example, despite women comprising the majority of the healthcare workforce, they hold disproportionately fewer senior leadership positions. Similarly, racial and ethnic minorities are underrepresented in executive roles, limiting the generalizability of findings. Research specifically targeting leadership styles among these groups and their unique impacts on safety culture is urgently needed.

Future reviews could broaden database coverage, include qualitative and mixed-methods studies, and enhance transparency in search and appraisal methods. Expanding the scope to diverse healthcare settings and professionals beyond nursing may also improve the generalizability and depth of findings. Addressing these gaps will better inform culturally sensitive, context-tailored leadership development interventions aimed at fostering robust patient safety cultures across all healthcare environments.

### Implications for future research

Future studies should examine the long-term impacts of varied leadership styles particularly transformational and ethical leadership style on patient safety in different healthcare settings. The study may require a longitudinal study to expedite examination of the effects of leadership in different healthcare settings such as acute care units, psychiatric wards, and nursing homes. The research will assist in understanding how sustained leadership practices shape patient safety outcomes over time based on varied care settings and patient demographics.

Additional research is required on the indirect impacts of leadership styles on patient culture such as role of patient safety initiatives mediating leadership behaviors and patient care outcomes. The studies can examine elements such as organizational support, nurse empowerment, and job demands that affect patient safety.

This study also suggests to prioritize longitudinal and experimental designs to delineate causal pathways and sustainment of leadership effects. Expanding the scope beyond nursing and inpatient settings, standardizing measurement tools, and incorporating objective outcome metrics that will strengthen the evidence base. Exploring cultural moderators of leadership effectiveness can inform globally applicable and contextually relevant leadership models.

Given the limitation of limited databased coverage in this study Future reviews could broaden database coverage, require inclusive, diversely representative samples, include qualitative and mixed-methods studies, and enhance transparency in search and appraisal methods, incorporating robust theoretical models to strengthen the interpretive clarity and scientific foundation, thereby advancing the field through hypothesis-driven investigation and facilitating evidence-based leadership interventions in healthcare settings. These could provide essential insights into the processes, challenges, and contextual moderators of leadership’s impact on safety culture that quantitative metrics alone might not capture. A dedicated qualitative or mixed-methods systematic review, or a meta-synthesis, could enhance understanding of staff experiences, organizational dynamics, and cultural influences, thereby informing more nuanced and effective leadership development programs in healthcare.

## Conclusion

This systematic review provides compelling evidence that nursing leadership styles play a critical role in shaping patient safety culture within healthcare settings globally. Transformational leadership consistently emerges as the most effective style, significantly enhancing dimensions such as teamwork, communication openness, organizational learning, and positive safety perceptions. Ethical leadership also evidences important contributions by fostering moral courage, ethical climates, and staff satisfaction. Transactional leadership offers more limited but complementary benefits by supporting short-term goals and operational clarity.

Healthcare organizations should prioritize embedding transformational and ethical leadership development into nursing leadership training and ongoing professional development. Such efforts should emphasize motivational communication, individualized support, ethical decision-making, and resilience-building to adapt to increasingly complex healthcare environments. Integrating leadership evaluation with safety performance metrics and sustained mentorship programs promises to embed and sustain these positive culture shifts.

Despite these insights, substantial evidence gaps remain. The predominance of cross-sectional study designs limits causal conclusions, necessitating future research to adopt longitudinal and intervention methodologies for elucidating leadership’s sustained impact. Understanding leadership’s mechanistic pathways via mediators such as psychological safety, communication patterns, and power dynamics remains nascent. Moreover, underrepresented leadership populations—including physicians, allied health professionals, and diverse demographic groups—are insufficiently studied, constraining the generalizability of findings across healthcare contexts.

Consequently, future research agendas should prioritize mixed-methods and intersectional approaches informed by robust theoretical models like High Reliability Organization Theory. Expanding inquiry beyond nursing to interdisciplinary healthcare teams will provide more comprehensive, nuanced insights guiding effective leadership strategies tailored to varied organizational and cultural contexts.

## Data Availability

The data used to support the findings of this study are from previously reported studies, which has been cited.
